# Floating Knee Injury Associated with Patellar Tendon Rupture: A Case Report and Review of Literature

**DOI:** 10.1155/2012/913230

**Published:** 2012-02-28

**Authors:** Singaravadivelu Vaidyanathan, Jagannath Panchanathan Ganesan, Mugundhan Moongilpatti Sengodan

**Affiliations:** ^1^Stanley Medical College, Chennai, Tamilnadu, India; ^2^ESI Hospital, KK Nagar, Chennai, Tamilnadu, India; ^3^Department of Orthopaedics, Coimbatore Medical College, Tamilnadu, Coimbatore 641036, India

## Abstract

Floating knee injuries are frequently associated with other concomitant injuries to the ipsilateral limb or other parts of body of which injury to the ipsilateral knee ligaments carries significance for various reasons. A middle-aged man sustained a floating knee injury following RTA. DCS fixation by bridge plating technique for the distal femur and lateral buttress plating by MIPO technique for proximal tibia were planned and executed under spinal anesthesia with image intensifier. In addition, there were patellar tendon rupture along with avulsion of VMO from the medial border of patella and torn MPFL, which we have missed initially. To the best of our knowledge no similar case has been reported in English literature so far. We have reviewed the literature and proposed a different interpretation of Blake and McBride classification.

## 1. Introduction

Ipsilateral fracture shafts of femur and tibia cause a floating knee injury. They are almost always due to high-energy trauma. Hence, they are frequently associated with other concomitant injuries to the ipsilateral limb or other parts of body of which injury to the ipsilateral knee ligaments carries significance for various reasons. We are reporting a case of floating knee injury associated with rupture of patellar tendon, vastus medialis obliques (VMOs), and medial patellofemoral ligament (MPFL). To the best of our knowledge, no similar case has been reported in English literature so far.

## 2. Case Report

**Figure 1:**
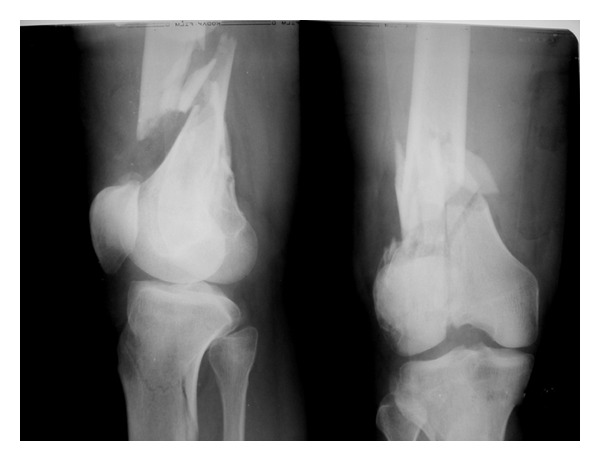
Preoperative X-ray of the right knee AP and lateral views showing fracture distal femur and proximal tibia.

A male patient aged 35 years presented to our emergency OPD following RTA, who while riding a two-wheeler was hit by another. The front bumper hit his knee. On examination, there were no external injury. There were swelling, tenderness, and deformity of distal thigh, knee, and proximal leg on the right side. There was no distal neurovascular deficit. X-ray of the right knee revealed fracture distal femur OTA A3 and fracture proximal tibia OTA A2 (Figure 1). 

**Figure 2:**
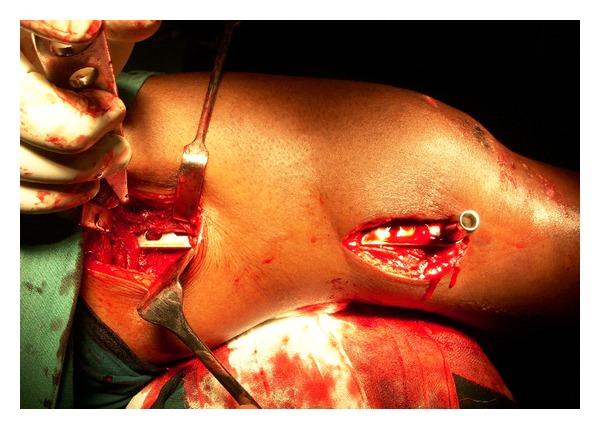
Fracture distal femur being fixed with DCS by MIPO.

According to Blake and McBryde classification, it can be classified as type I floating knee injury. DCS fixation by bridge plating technique for the distal femur and lateral buttress plating by MIPO technique for proximal tibia were planned and executed under spinal anesthesia with image intensifier (Figure 2).

 While closing the surgical wounds, the knee was kept in flexed position, and the second author noticed the absence of patellar tendon prominence.

**Figure 3:**
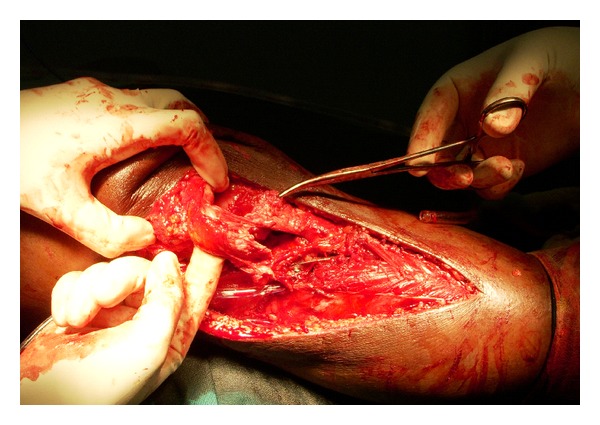
Intraoperative picture showing torn ends of patellar tendon.

**Figure 4:**
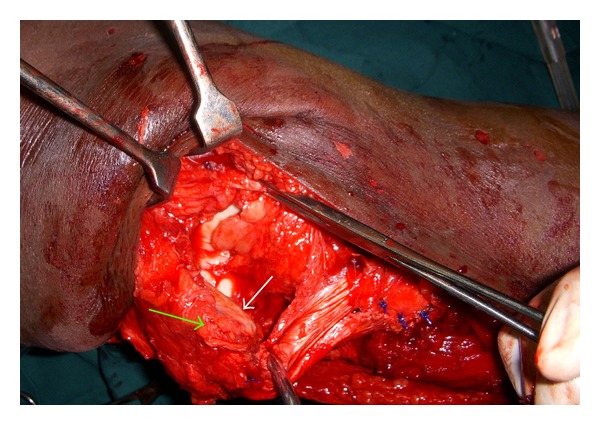
Patella tilted 90° laterally (white arrow points to articular surface) to show the bare medial border (green arrow) from where VMO and MPFL were torn.

 The incision was extended to expose the patella and its tendon. We were surprised to find the torn patellar tendon (Figure 3) along with avulsion of VMO from the medial border of patella and torn MPFL (Figure 4). 

**Figure 5:**
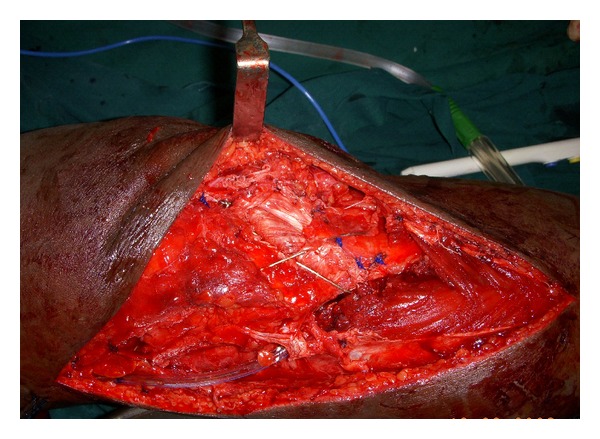
Patellar tendon repaired and protected with a figure of 8 wiring.

 All the three injured structures were repaired. The patellar tendon was protected by a figure of 8 tension band through patella and tibial tuberosity (Figure 5). 

**Figure 6:**
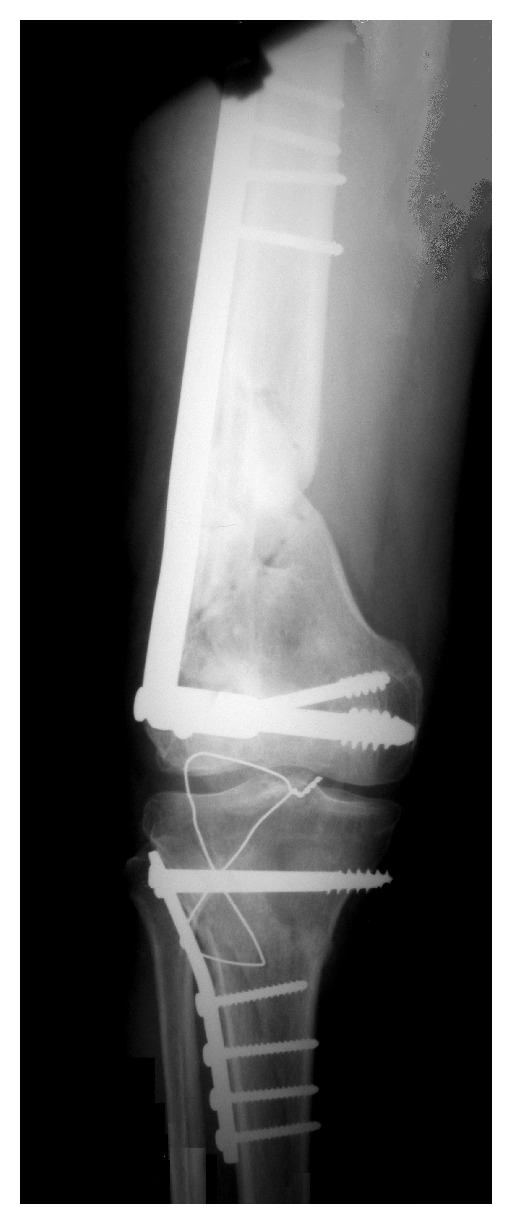
3-month followup X-ray of the right knee AP showing fracture union.

**Figure 7:**
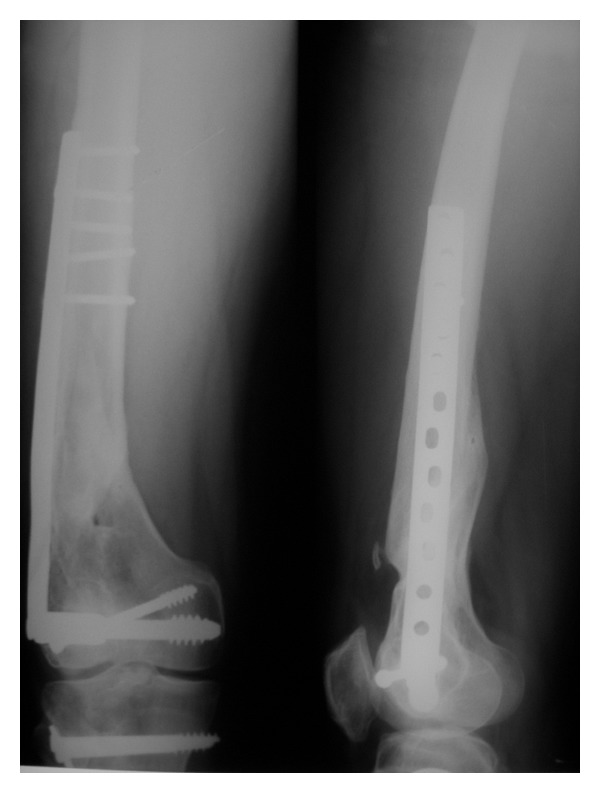
X-ray right femur AP and lateral views at 2-year followup showing fracture union.

**Figure 8:**
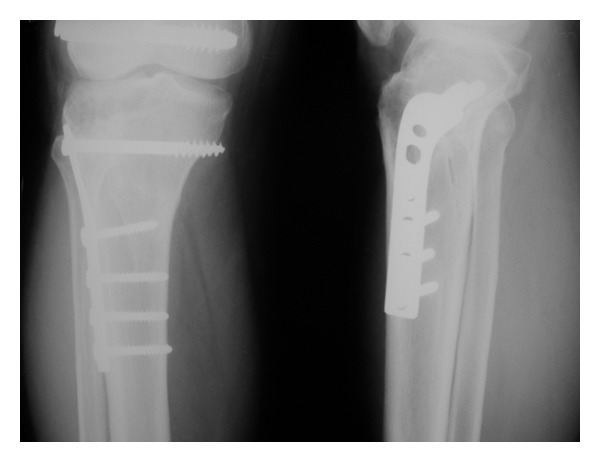
X-ray right leg AP and lateral views at 2-year followup showing fracture union.

**Figure 9:**
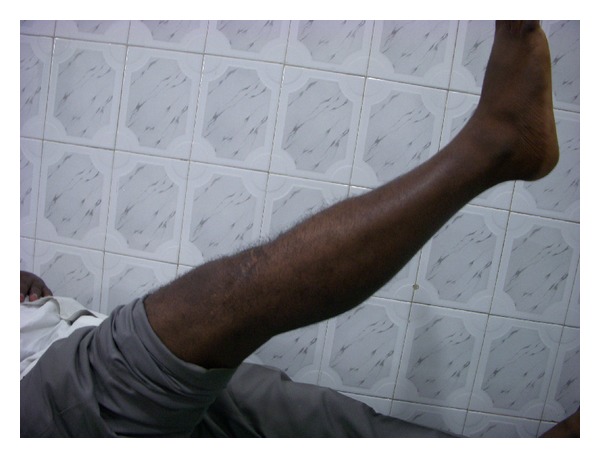
Clinical picture at 2-year followup after figure of 8 wire removal showing active SLR.

**Figure 10:**
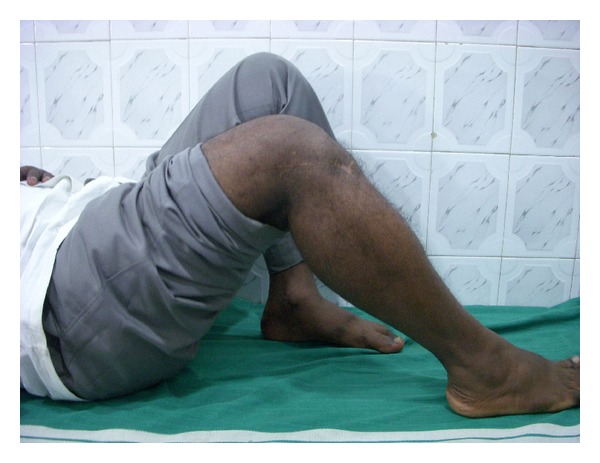
Clinical picture at 2-years followup after figure of 8 wire removal showing knee flexion up to 100°.

 Limb was supported with a long knee brace, and a non-weight-bearing mobilization was started from day 2. Weight bearing was allowed after radiological union of both the fractures after 3 months. 90 degrees of knee flexion was achieved by 6 months and the figure of 8 TBW was removed at that time (Figure 6). At 2-year followup, the patient has got a stable knee with the ROM of 0 to 100 degrees without any extensor lag or lateral patellar subluxation (Figures 7, 8, 9, and 10).

## 3. Discussion

Floating knee is a term coined by Blake and McBryde to describe ipsilateral fractures of femur and tibia [1]. Floating knee injury is often due to high-velocity trauma. Hence, it is usually associated with multiple injuries to the same limb or to other parts of the body. Rethnam et al. emphasized a thorough secondary survey to assess the associated injuries. He described floating knee injury as “more than what meets the eye.” The grossly deformed limb that one encounters in the floating knee can act as a major distracting factor, and it is not unusual to miss other significant injuries [2, 3].

 In our case, we would have missed the associated injuries to the patellar tendon, VMO, and MPFL if we had sutured the surgical wound with the knee in extension.

 Of the associated injuries to floating knee injuries, involvement of ipsilateral knee ligaments is particularly significant because they are most often the missed ones and contributed to poorer outcome in most of the studies [2–7].

 Szalay et al. observed demonstrable knee ligament laxity in 18 out of 34 cases (53%) of floating knee injury at followup, and 6 (18%) of them complained of instability. Injury to ACL was the most common finding. And they advocated careful assessment of the knee in all cases of fracture femur and especially when tibia is also fractured [4].

 In a retrospective study by van Raay et al., 15 out of 47 (31%) patients with ipsilateral fractures of femoral and tibial diaphyses proved to have instability of the knee at the time of followup of which only 3 cases had been diagnosed to have the knee ligament injury at the time of initial treatment [5].

 Schiedts et al. encountered late diagnosis of ipsilateral knee ligamentous injury: anterior and posterior in three and lateral isolated in one. All the four patients out of 18 in their series had poor functional outcome. They concluded that knee instability is the major cause of poor results [6].

Kao et al., in their retrospective study, noticed 44 cases of knee ligament injury out of 419 cases (10.5%) of floating knee injury, 23 PCL, and 18 ACL [8].

In a series of 29 patients reported by Rethnam et al., they encountered knee ligament injury in 4 patients (2 ACL, 1 PCL, and 1 medial meniscus). All the ligamentous injuries were detected and repaired at the time of fracture fixation itself [2, 3].

 Dickob and Mommsen observed ipsilateral knee ligamentous damage in 37.2% of 43 cases with extra-articular fractures near the knee. They opined that restoration of knee joint motion is more important than joint stability. And therefore, ligamentous repair has to be performed secondarily if necessary [7].

 In our case, there was a midsubstance tear of the patellar tendon. In addition, there was avulsion of VMO from the medial border of the patella and midsubstance tear of MPFL.

 We could not find a case of floating knee injury in association with rupture of patellar tendon, VMO, and MPFL being reported in English literature even after extensive search.

 The classification system by Blake and McBryde [1] was found to be the most comprehensive and widely used (Table 1). 

 Our case is classified as type I radiologically. But we would like to propose a different interpretation of the classification.

Karlstrom and Olerud score was used for assessing the outcome in almost all the studies [9].

 In our case, the functional outcome was good according to K_O score.

There has been consistent correlation between poor clinical outcome and the associated ligament injury in a case of floating knee injury more than any other individual variability in almost all the studies [2, 3, 10, 11].

Yokoyama et al. stated the involvement of knee joint and soft tissue injury in the tibia as the major risk factors responsible for the poor outcome in floating knee injuries in a multivariate analysis in 68 cases [10]. Hung et al. also found that the intra-articular knee involvement is the most important factor contributing to poorer outcome [11].

Rethnam et al. had poor functional outcome in 3 patients out of 4, those who had knee ligament injury even after repair [2, 3]. Schiedts et al. reported poor outcome in all the 4 cases with knee ligament injury [6].

As per the description, Blake and McBryde classification type II A denotes fracture involving the knee joint. But even if the fracture is juxta-articular and not involving the knee joint, if there is an associated knee ligamentous injury, it significantly changes the outcome. Hence, we feel the radiological classification system should be revised intraoperatively, and if there is a knee ligament injury, it can be classified under type II A.

## 4. Conclusion

We have presented this case for its rarity and emphasizing the importance of thorough secondary survey for knee ligamentous injury which is quite often missed. We have also reviewed the literature to bring out the different perspective of the Blake and McBryde classification system when there is an associated knee ligament injury in a case of floating knee injury.

## Figures and Tables

**Table 1:** Blake and McBryde classification for floating knee injuries.

Type 1—true floating knee	The knee joint is isolated completely and not involved, with either shaft fractured
Type 2—variant floating knee	Involves one or more joints with either shaft fractured
Type 2A	The knee joint alone is involved
Type 2B	Involves the hip or ankle joints
